# Association between serum iron levels and atherosclerotic cardiovascular diseases among American older adults: a cross-sectional study based on the National Health and Nutrition Examination Survey, 2009–2018

**DOI:** 10.3389/fnut.2024.1457236

**Published:** 2024-09-25

**Authors:** Xiaochen Yu, Min Li, Bingxing Chen, Yuan Qi, Xiuru Guan

**Affiliations:** Department of Laboratory Diagnostics, The First Affiliated Hospital of Harbin Medical University, Harbin, Heilongjiang, China

**Keywords:** serum iron levels, ASCVD, NHANES, BMI, older adults

## Abstract

**Background:**

There is controversy regarding the relationship between serum iron levels and atherosclerotic cardiovascular disease (ASCVD).

**Objective:**

To investigate the relationship between serum iron levels and ASCVD among older adults using data from the 2009–2018 National Health and Nutrition Examination Survey (NHANES).

**Methods:**

We performed a cross-sectional analysis involving 8,682 participants aged 60 years and older, with complete data on serum iron levels and confirmed ASCVD status, sourced from the 2009–2018 National Health and Nutrition Examination Survey (NHANES). Multivariable logistic regression models were used to examine the association between serum iron levels and ASCVD. To assess the consistency of this association across different demographic groups, subgroup analyses, and interaction tests were performed.

**Results:**

The group with the highest serum iron levels (fourth quartile, 100–369 μg/dL) exhibited several distinct characteristics: they were the youngest on average (69.57 ± 6.91 years), had the highest proportion of males (61.42%), and the highest hemoglobin levels (14.43 ± 1.33 g/dL). This group also showed the lowest iron supplement usage (19.71 ± 12.85 mg/30 days), white blood cell counts (6.73 ± 2.41 1,000 cells/μL), and serum creatinine levels (0.98 ± 0.45 mg/dL). Moreover, they had higher levels of education and income, a higher likelihood of being married, and a lower body mass index (BMI). Additionally, they had significantly lower rates of diabetes, hypertension, stroke, and heart attacks (all *p* < 0.05). After adjusting for potential confounders, a linear relationship between serum iron levels and ASCVD was initially observed (OR = 0.97; 95% CI, 0.95–0.99, *p* < 0.05). However, further analysis using a two-part logistic regression model with an inflection point at 131 μg/dL revealed more nuanced results. For serum iron levels below 131 μg/dL, each 10 μg/dL increase was associated with a 4% decrease in the odds of ASCVD (OR = 0.96; 95% CI, 0.93–0.98, *p* < 0.001). Conversely, for serum iron levels above 131 μg/dL, each 10 μg/dL increase corresponded to a 1% increase in the odds of ASCVD, though this finding was not statistically significant (OR = 1.01; 95% CI, 0.98–1.08, *p* > 0.05).

**Conclusion:**

In the US elderly population, serum iron levels are negatively associated with ASCVD, particularly when serum iron levels are below 131 μg/dL.

## 1 Introduction

In recent decades, advancements in drug treatments, medical devices, and interventional techniques have significantly reduced the risk and mortality associated with atherosclerotic cardiovascular disease (ASCVD) (1). Nevertheless, cardiovascular diseases (CVDs) remain the leading cause of death globally and a serious threat to human health (2). Atherosclerosis (AS) plays a significant role in the development of CVDs (3). The formation of AS is a complex process involving lipids, inflammation, oxidative stress, among other factors. Research suggests that increased ROS likely contributes to the development of ASCVD (4). The degree of oxidation is directly correlated with the severity of AS (5). Certain oxidative biomarkers, such as oxidized low-density lipoprotein (ox-LDL) and advanced glycation end products (AGEs), have been reported as independent predictors of ASCVD in large observational cohort studies (6, 7).

Iron, an essential micronutrient and the most abundant transition metal in the human body plays a crucial role in the synthesis, transport, and metabolism of various substances (8). Iron predominantly exists in two forms: non-heme ferric ions (Fe^3+^) and heme ferrous ions (Fe^2+^) (9). These two forms are interconvertible, with Fe^3+^ and Fe^2+^ capable of transitioning between their ferric and ferrous states, respectively.

Iron deficiency impairs exercise capacity by disrupting cardiac and skeletal muscle function, altering mitochondrial activity, and weakening cardiac energy dynamics (10, 11). However, excessive iron accumulation can also be detrimental, as it promotes the production of hydroxyl radicals through the Haber–Weiss and Fenton reactions. This leads to oxidative stress and subsequent damage to cellular components, including lipids, proteins, and DNA (12, 13). Several studies have shown that excessive dietary iron intake is associated with the progression of CVDs, including stroke and coronary heart disease (14–16).

Although some studies suggest that increased iron stores are associated with a higher risk of developing CADs, the relationship between serum iron levels and ASCVD remains controversial in clinical observations (17, 18). Consequently, it is essential to further investigate the relationship between serum iron levels and ASCVD in large population cohorts.

## 2 Materials and methods

### 2.1 Survey description

All cross-sectional data for this study were obtained from the National Health and Nutrition Examination Survey (NHANES). Conducted by the National Center for Health Statistics (NCHS), NHANES is an ongoing survey that employs a multistage, stratified probability sampling method every two years. It provides a nationally representative snapshot of the nutritional status and health risk factors among the non-institutionalized civilian population in the United States. Demographic information, dietary intake, and health status data were collected through in-home interviews. Physical examinations and laboratory tests were conducted in mobile examination centers (MECs) specifically designed for this purpose. We retrieved data including demographic details, dietary records, physical examination findings, laboratory measurements, and questionnaire responses. All participants in the NHANES study provided informed consent before their participation. Comprehensive details about the study protocols, experimental design, and data are publicly available at the following link: https://www.cdc.gov/nchs/nhanes/.

**FIGURE 1:**
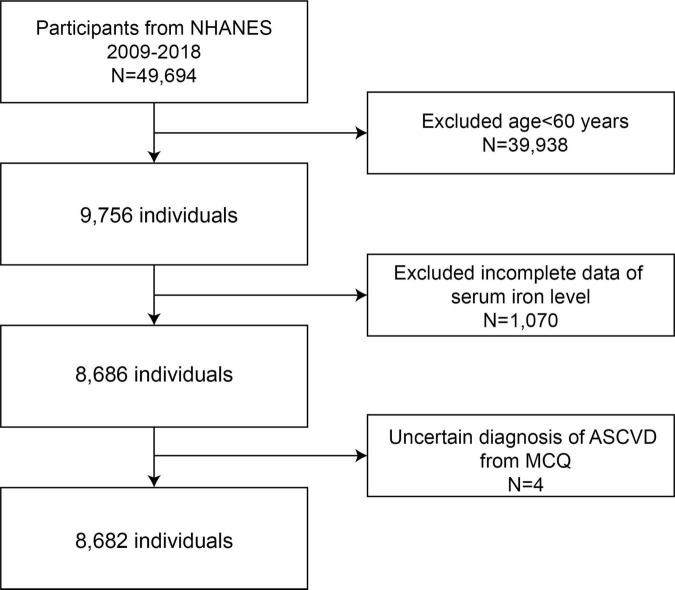
Flow chart of the sample selection from NHANES 2009–2018. NHANES, National Health and Nutrition Examination Survey.

We utilized five NHANES cycles from 2009 to 2018 to assess the association between serum iron levels and ASCVD. Initially, a total of 49,694 participants were enrolled in the study. After applying exclusion criteria, we included 8,682 eligible participants in the final study. The exclusion criteria were as follows: age < 60 years (*n* = 39,938), missing serum iron data (*n* = 1,070), and uncertain diagnosis of ASCVD from the Multiple Choice Question (MCQ) (*n* = 4). Ultimately, 8,682 participants were included for further analysis (Figure 1).

In complex studies, sampling weights are typically used to ensure that the data accurately reflects the population and is free from bias. However, adjusting these weights for regression analysis can introduce significant bias and potentially reduce the precision of the findings. Therefore, we chose to present our results without applying sampling weights to avoid over-adjustment.

### 2.2 Exposure and outcome variables

Serum iron levels were the primary exposure variable of interest. Serum specimens were processed, stored, and shipped to the Collaborative Laboratory Services for analysis. Detailed instructions on specimen collection and processing are provided in the NHANES Laboratory/Medical Technologists Procedures Manual (LPM). Vials were stored frozen at −30°C as specified in the NHANES protocol until they were shipped to the National Center for Environmental Health for testing. In this process, iron is released from transferrin by acetic acid, reduced to the ferrous state by hydroxylamine and thioglycolate, and immediately complexed with the FerroZine Iron Reagent. The system monitored the absorbance at 560 nm at fixed intervals, with the absorbance change being directly proportional to the iron levels in the sample.

The outcome was ASCVD, defined according to the 2013 guidelines published by the American College of Cardiology (ACC) and the American Heart Association (AHA) concerning the management of blood cholesterol to mitigate the risk of atherosclerotic cardiovascular disease in adults (19). ASCVD was characterized by the presence of at least one diagnosis among coronary heart disease, angina, heart attack, or stroke (20). We defined ASCVD by a synthesis of self-reported physician diagnoses and standardized medical condition questionnaires completed during personal interviews, as detailed in Supplementary Table 1.

### 2.3 Covariates assessment

We included several covariates that could influence the association between serum iron levels and ASCVD in participants. These covariates were age (years), gender (male/female), race/ethnicity (Non-Hispanic White/Non-Hispanic Black/Mexican American/Other races), iron supplements, hemoglobin (HGB), white blood cell (WBC), serum creatinine (SCR), education (less than high school/high school graduate or equivalent/some college or AA degree/college graduate or above), income to poverty ratio (PIR, ≤ 1/> 1), marital status (married/never married/living with a partner/others). BMI was calculated as weight divided by height squared (kg/m^2^) and categorized as normal weight (< 25.0 kg/m^2^), overweight (25.0–29.9 kg/m^2^), and obese ( ≥ 30.0 kg/m^2^). According to NHANES, a person is identified as a smoker if they have smoked more than 100 cigarettes in their lifetime. The covariates also included diseases such as hypertension, hyperlipidemia, and diabetes, with diagnostic criteria detailed in Supplementary Table 1. To handle missing data in the iron supplements, a regression model was used to impute the missing values. To ensure the accuracy of our findings and minimize bias, we adjusted for hyperlipidemia, hypertension, and diabetes in our analysis, recognizing them as independent risk factors for ASCVD (21).

### 2.4 Statistical analysis

Statistical analysis was performed using R version 3.4.3^1^ (The R Foundation) and Empower software^2^ (X&Y Solutions, Inc., Boston, MA). Participants were stratified into four groups based on serum iron quartiles: Q1 (2–60 μg/dL), Q2 (61–78 μg/dL), Q3 (79–99 μg/dL), and Q4 (100–369 μg/dL). Continuous variables are presented as mean ± standard deviation, while categorical variables are presented as frequencies and percentages. For continuous variables, *p*-values were calculated using the Kruskal-Wallis test. Fisher’s exact test was employed to determine *p*-values for categorical variables when the expected count was less than 10.

We examined the relationship between serum iron levels and the odds of ASCVD, focusing on each 10-fold increase in serum iron levels. Multifactorial logistic regression was used to assess the association between serum iron and ASCVD across three different models. Model 1 was unadjusted for covariates. Model 2 was adjusted for age, gender, and race/ethnicity. Model 3 was further adjusted for iron supplements, HGB, WBC, SCR, education, PIR, marital status, BMI, smoking, diabetes, hyperlipidemia, and hypertension. Univariate analysis was conducted to evaluate the relationship between each covariate and ASCVD within the fully adjusted model. Smoothed regression curves were utilized to evaluate the linear association between the serum iron levels and ASCVD in the context of Model 3. For subgroup analyses, we assessed the association of serum iron levels with ASCVD across various effect modifiers, including gender, age (60–70; 70–80; > 80 years), race/ethnicity, iron supplements, HGB, WBC, SCR, education level, marital status, BMI (< 25.0; 25.0–30.0; > 30.0 kg/m^2^), PIR, smoking status (yes/no), diabetes (yes/no), hyperlipidemia (yes/no), and hypertension (yes/no). These stratification factors were considered as potential effect modifiers to test for heterogeneity in the associations across subgroups.

## 3 Results

### 3.1 Baseline characteristics of selected participants

A total of 8,682 participants meeting the inclusion criteria were included in the study, with a mean age of 69.99 ± 6.96 years, and 49.61% were male. Baseline characteristics are presented according to serum iron quartiles: Q1 (2–60 μg/dL), Q2 (61–78 μg/dL), Q3 (79–99 μg/dL), and Q4 (100–369 μg/dL) (Table 1). Significant differences were observed among the serum iron quartiles in demographic, clinical, and lifestyle factors such as age, gender, iron supplements, HGB, WBC, SCR, education, PIR, and health outcomes including diabetes, hypertension, stroke, and heart attack (*p* < 0.05). Participants in the Q4 group were more likely to be younger, male, Non-Hispanic White, college graduate or above, PIR > 1, the lowest iron supplements, WBC, SCR and BMI, and the highest HGB. Similarly, participants in the Q4 group had the lowest odds of diabetes, hypertension, stroke, and heart attack. However, no significant differences were observed among the quartiles regarding iron supplements, smoking, hyperlipidemia, coronary heart disease, and angina.

**TABLE 1:** Baseline characteristics of participants, from National Health and Nutrition Examination Survey (NHANES) 2009–2018, total (8,682).

Characteristics	Serum iron levels					*P*-value
	Total	Q1 (*N* = 2,056)	Q2 (*N* = 2,176)	Q3 (*N* = 2,227)	Q4 (*N* = 2,227)	
Age, (years)	69.99 ± 6.96	70.28 ± 6.98	70.06 ± 6.97	70.08 ± 6.96	69.57 ± 6.91	0.007
Iron supplements, (mg/30 days)	21.18 ± 13.81	23.57 ± 14.84	21.26 ± 3.53	20.35 ± 13.73	19.71 ± 12.85	< 0.001
HGB, (g/dL)	13.75 ± 1.47	12.93 ± 1.53	13.64 ± 1.29	13.95 ± 1.33	14.43 ± 1.33	< 0.001
WBC, (1,000 cells/μL)	7.14 ± 5.16	7.79 ± 9.22	7.22 ± 3.75	6.88 ± 2.31	6.73 ± 2.41	< 0.001
SCR, (mg/dL)	1.02 ± 0.57	1.08 ± 0.69	1.02 ± 0.51	1.00 ± 0.62	0.98 ± 0.45	< 0.001
Gender, (%)						< 0.001
Male	49.61	39.93	46.48	49.80	61.42	
Female	50.39	60.07	53.52	50.20	38.53	
Race/ethnicity, (%)						< 0.001
Non-Hispanic White	46.35	40.61	45.70	48.32	50.31	
Non-Hispanic Black	20.63	28.06	24.05	18.46	12.59	
Mexican American	12.43	11.43	12.28	12.48	13.44	
Other races	20.59	19.89	17.97	20.75	23.65	
Education level, (%)						< 0.001
< High school	29.01	33.03	29.66	28.24	25.45	
High school graduate	23.11	24.46	22.71	22.54	22.80	
Some college	26.80	25.54	27.77	26.54	27.29	
College graduate or above	20.84	16.68	19.54	22.41	24.37	
PIR, (%)						< 0.001
≤ 1	72.54	22.65	18.26	17.77	15.99	
> 1	16.55	77.35	81.74	82.23	84.01	
Marital status (%)						< 0.001
Married	54.57	48.88	54.90	53.88	60.21	
Never married	5.47	5.45	6.85	5.03	4.59	
Living with a partner	2.74	1.70	3.17	2.92	3.10	
Other	37.21	43.97	35.08	38.17	32.1	
BMI, kg/m^2^	29.27 ± 6.34	30.35 ± 7.12	29.78 ± 6.47	28.89 ± 6.04	28.15 ± 5.50	< 0.001
BMI, kg/m2, (%)						< 0.001
Normal weight	24.86	21.94	22.40	26.69	28.07	
Over weight	36.23	31.93	35.76	36.47	40.37	
Obesity	38.91	46.13	41.84	36.83	31.55	
Smoking, (%)						0.212
Yes	50.25	49.56	50.23	48.85	52.36	
No	49.63	50.34	49.72	50.97	47.51	
Diabetes, (%)						< 0.001
Yes	35.27	41.54	36.87	33.32	29.86	
No	64.73	58.46	63.13	66.68	70.14	
Hyperlipidemia, (%)						0.756
Yes	82.93	82.64	83.45	83.25	82.37	
No	17.07	17.36	16.55	16.75	17.63	
Hypertension, (%)						< 0.001
Yes	71.32	76.99	72.37	69.82	66.55	
No	28.6829	23.01	27.63	30.18	33.45	
Stroke, (%)						< 0.001
Yes	8.35	9.82	9.65	7.41	6.65	
No	91.52	90.03	90.17	92.50	93.08	
Coronary heart disease, (%)						0.191
Yes	10.12	10.65	10.98	10.19	8.71	
No	89.10	88.42	88.10	89.13	90.53	
Angina, (%)						0.079
Yes	5.38	6.61	5.38	5.21	4.40	
No	94.02	92.61	93.93	94.34	94.93	
Heart attack, (%)						0.037
Yes	9.54	10.41	10.89	8.31	8.62	
No	90.24	89.30	88.83	91.51	91.06	

Mean ± SD for continuous variables: *p*-values were calculated using the weighted linear regression model. For categorical variables (%), *p*-values were calculated using the weighted chi-square test. HGB, hemoglobin; WBC, white blood cell; SCR, serum creatinine; PIR, the ratio of income to poverty; BMI, body mass index; Q, quartile.

### 3.2 Association of serum iron levels with ASCVD

Our results demonstrated that higher serum iron levels were consistently associated with a lower likelihood of ASCVD across all models, including the crude model (OR = 0.96; 95% CI, 0.94–0.97, *p* < 0.0001), the minimally adjusted model (OR = 0.94; 95% CI, 0.93–0.96, *p* < 0.0001), and the fully adjusted model (OR = 0.97; 95% CI, 0.95–0.99, *p* < 0.05). In a fully adjusted model, each 10-fold increase in serum iron levels was associated with a statistically significant 3% decrease in the odds of ASCVD (OR = 0.97; 95% CI, 0.95–0.99, *p* < 0.05). When serum iron levels were categorized into quartiles, participants in the highest quartile (Q4) had an 25% lower risk of ASCVD compared to those in the lowest quartile (Q1) (OR = 0.75; 95% CI, 0.63–0.90, *p* < 0.05). Participants in the third quartile (Q3) had a 13% lower odds of ASCVD compared to the lowest quartile (Q1), although this result was not statistically significant (OR = 0.87; 95% CI, 0.74–1.04, *p* > 0.05). Similarly, the difference between participants in the second quartile (Q2) and the lowest quartile (Q1) was not statistically significant (OR = 1.02; 95% CI, 0.86–1.20, *p* > 0.05) (Table 2).

**TABLE 2:** Association between 10-fold serum iron levels and ASCVD.

Exposure	Model 1 [OR (95% CI)]	Model 2 [OR (95% CI)]	Model 3 [OR (95% CI)]
10-fold serum iron levels (continuous)	0.96 (0.94, 0.97) < 0.0001	0.94 (0.93, 0.96) < 0.0001	0.97 (0.95, 0.99) 0.0014
**10-fold serum iron levels (quartile)**			
Quartile 1	Reference	Reference	Reference
Quartile 2	0.96 (0.84, 1.11) 0.5940	0.92 (0.79, 1.06) 0.2378	1.02 (0.86, 1.20) 0.8129
Quartile 3	0.80 (0.69, 0.93) 0.0025	0.74 (0.64, 0.86) < 0.0001	0.87 (0.74, 1.04) 0.1196
Quartile 4	0.70 (0.60, 0.81) < 0.0001	0.61 (0.52, 0.71) < 0.0001	0.75 (0.63, 0.90) 0.0019
*P* for trend	< 0.0001	< 0.0001	< 0.001

Model 1, no covariates were adjusted. Model 2, age, gender, race/ethnicity were adjusted. Model 3, age, gender, race/ethnicity, HGB, WBC, SCR, education, PIR, marital status, BMI, smoking, diabetes, hyperlipidemia, hypertension were adjusted. HGB, hemoglobin; WBC, white blood cell; SCR, serum creatinine; PIR, the ratio of income to poverty; BMI, body mass index.

**FIGURE 2:**
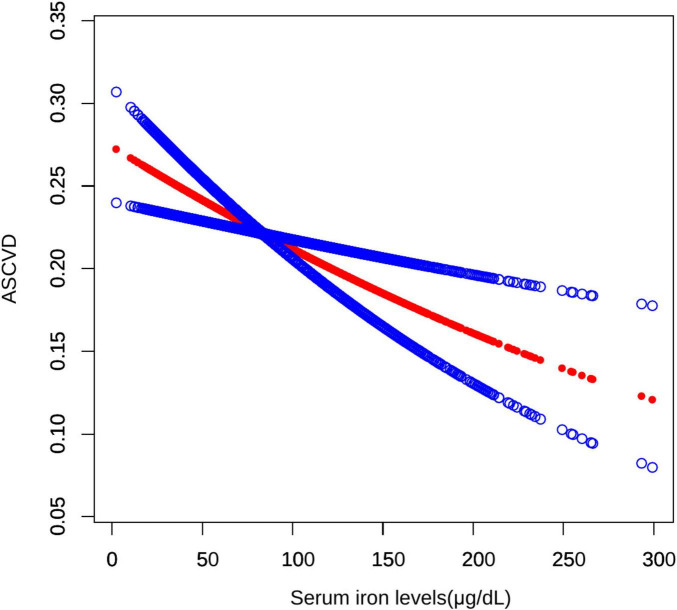
Smooth curve fitting for serum iron levels and ASCVD. A non-linear relationship between serum iron levels and ASCVD was identified using a generalized model.

Initially, the graph illustrates a linear relationship between serum iron levels and ASCVD, showing that as serum iron levels increase, the odds of ASCVD decrease, indicating a significant negative association. Specifically, each 10-fold increase in serum iron levels was associated with a 3% decrease in the odds of ASCVD (OR = 0.97; 95% CI, 0.95–0.99, *p* < 0.05). In the subsequent threshold effect analysis, an inflection point was identified at 131 μg/dL (Table 3). When serum iron levels were below 131 μg/dL, each 10 μg/dL increase was associated with a 4% decrease in the odds of ASCVD (OR = 0.96; 95% CI, 0.93–0.98, *p* < 0.001). However, when serum iron levels exceeded 131 μg/dL, each 10 μg/dL increase was associated with a 1% increase in the odds of ASCVD, although this result was not statistically significant (OR = 1.01; 95% CI, 0.98–1.08, *p* > 0.05) (Figure 2).

**TABLE 3:** Results of the threshold effect analysis for serum iron levels and ASCVD.

Models	Adjusted OR (95% CI), *P*-value
Standard linear model	0.97 (0.95, 0.98) 0.0014
**Two-part logistic regression model**	
Inflection point	131
Serum iron concentration < 131	0.96 (0.93, 0.98) < 0.001
Serum iron concentration ≥ 131	1.01 (0.98, 1.08) 0.7176
Log likelihood ratio	0.172

### 3.3 Subgroup analysis

**FIGURE 3:**
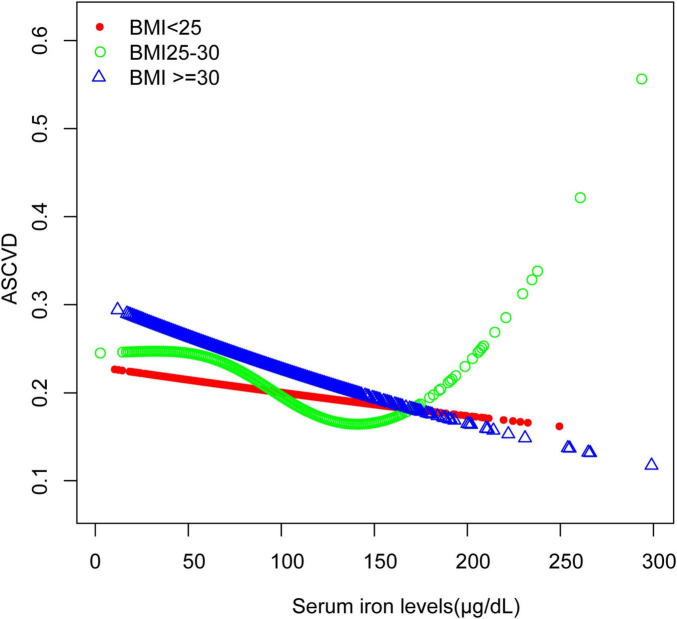
Smooth curve fitting for serum iron levels and ASCVD stratified by BMI. The relationship between serum iron levels and ASCVD was identified using a generalized model, stratified by BMI.

The association between serum iron levels and ASCVD was further analyzed using both subgroup analysis and an interaction test. Interaction tests indicated that none of the evaluated factors significantly modified the relationship between serum iron levels and ASCVD (all *p* for interaction > 0.05) (Table 4). However, subgroup analysis revealed significant differences in factors like gender, BMI, and smoking, among others (Table 4). Notably, among participants with BMI of 25–30 kg/m^2^, the relationship between serum iron levels and ASCVD follows a typical U-shaped curve (Figure 3). For serum iron levels below 136 μg/dL, each 10 μg/dL increase is associated with a 6% decrease in the odds of ASCVD (OR = 0.94; 95% CI, 0.90–0.98, *p* < 0.05). Conversely, for serum iron levels above 136 μg/dL, each 10 μg/dL increase is associated with a 14% increase in the odds of ASCVD (OR = 1.14; 95% CI, 1.01–1.28, *p* < 0.05) (Table 5). This U-shaped pattern was not observed in participants with a BMI < 25 or > 30 kg/m^2^.

**TABLE 4:** Subgroup analysis of the association between serum iron levels and ASCVD.

Subgroup	*N*	ASCVD [OR (95% CI)]	*P* for interaction
Genders			0.6176
Male	3,777	0.97 (0.94, 0.99) 0.0140	
Female	3,803	0.96 (0.93, 0.99) 0.0154	
Age			0.7658
60 ≤ age < 70	3,886	0.97 (0.94, 1.00) 0.0369	
70 ≤ age < 80	2,283	0.97 (0.94, 1.01) 0.1548	
≥ 80	1,392	0.95 (0.91, 1.00) 0.0354	
Race/ethnicity, (%)			0.5886
Non-Hispanic White	3,620	0.97 (0.95, 1.00) 0.0597	
Non-Hispanic Black	1,540	0.93 (0.89, 0.99) 0.0126	
Mexican American	896	0.97 (0.91, 1.04) 0.3871	
Other races	1,505	0.96 (0.91, 1.01) 0.0912	
Education level, (%)			0.1374
< high school	2,139	1.00 (0.96, 1.04) 0.9672	
High school graduate	1,762	0.96 (0.92, 1.00) 0.0420	
Some college	2,065	0.94 (0.90, 0.98) 0.0057	
College graduate or above	1,595	0.95 (0.90, 1.00) 0.0521	
Marital status (%)			0.7067
Married	4,162	0.97 (0.95, 1.00) 0.0691	
Never married	390	0.92 (0.83, 1.02) 0.1185	
Living with a partner	194	0.93 (0.80, 1.08) 0.3506	
Other	2,815	0.97 (0.93, 1.00) 0.0363	
BMI			0.6740
< 24.9 kg/m^2^	1,879	0.98 (0.94, 1.02) 0.3096	
25–29.9 kg/m^2^	2,732	0.96 (0.93, 1.00) 0.0346	
≥ 30 kg/m^2^	2,950	0.96 (0.93, 0.99) 0.0132	
PIR, (%)			0.1911
≤ 1	1,396	0.99 (0.95, 1.03) 0.6348	
> 1	6,165	0.96 (0.94, 0.98) 0.0004	
Smoking, (%)			0.1745
Yes	3,842	0.96 (0.93, 0.98) 0.0016	
No	3,714	0.99 (0.95, 1.02) 0.3857	
Diabetes, (%)			0.1791
Yes	2,642	0.98 (0.95, 1.02) 0.3391	
No	4,919	0.96 (0.93, 0.98) 0.0013	
Hyperlipidemia, (%)			0.0901
Yes	6,287	0.96 (0.94, 0.98) 0.0005	
No	1,274	1.01 (0.96, 1.06) 0.7489	
Hypertension, (%)			0.5180
Yes	5,392	0.96 (0.94, 0.99) 0.0017	
No	2,169	0.98 (0.94, 1.02) 0.3221	

Age, iron supplements, HGB, WBC, SCR gender, race/ethnicity, education level, Marital status, PIR, BMI, smoking, diabetes, hyperlipidemia and hypertension were adjusted. HGB, hemoglobin; WBC, white blood cell; SCR, serum creatinine; PIR, the ratio of income to poverty; BMI, body mass index; ASCVD, atherosclerotic cardiovascular disease.

**TABLE 5:** Results of the threshold effect analysis for serum iron levels and ASCVD among participants with BMI 25–30 kg/m^2^.

Models	Adjusted OR (95% CI), *P*-value
Standard linear model	0.97 (0.93, 1.00) 0.0639
**Two-part logistic regression model**	
Inflection point	136
Serum iron concentration < 136	0.94 (0.90, 0.98) 0.0030
Serum iron concentration ≥ 136	1.14 (1.01, 1.28) 0.0303
Log likelihood ratio	0.008

## 4 Discussion

Both iron deficiency and iron overload can lead to the production of oxidative stress (22, 23), a key pathogenic mechanism in the development of ASCVD. However, the relationship between serum iron levels and ASCVD remains contentious in clinical observations. Our current research, based on a nationally representative cross-sectional analysis of 8,682 US adults, demonstrated a significant association between serum iron levels and ASCVD. Notably, we observed a negative relationship between serum iron levels and ASCVD (OR = 0.97; 95% CI, 0.95–0.97, *p* < 0.05). Using a two-part logistic regression model with an inflection point at 131 μg/dL, our analysis revealed a complex relationship between serum iron levels and ASCVD. For serum iron levels below 131 μg/dL, each 10 μg/dL increase in serum iron was associated with a 4% reduction in the odds of ASCVD, a statistically significant inverse relationship (OR = 0.96; 95% CI, 0.93–0.98, *p* < 0.001). For serum iron levels above 131 μg/dL, each 10 μg/dL increase was associated with a 1% increase in the odds of ASCVD, though this positive association was not statistically significant (OR = 1.01, 95% CI, 0.98–1.08, *p* > 0.05).

In participants with a BMI between 25 and 30 kg/m^2^, the relationship between serum iron levels and ASCVD followed a U-shaped curve (Figure 3). For these individuals, the inflection point was identified at 136 μg/dL. When serum iron levels were below 136 μg/dL, each 10 μg/dL increase in serum iron was associated with a 6% decrease in the odds of ASCVD (OR = 0.94; 95% CI, 0.90–0.98, *p* < 0.05). Conversely, for serum iron levels above 136 μg/dL, each 10 μg/dL increase was linked to a 14% increase in the odds of ASCVD (OR = 1.14; 95% CI, 1.01–1.28, *p* < 0.05). Individuals within this BMI range are at a critical point for factors such as oxidative stress, making them more susceptible to the effects of varying iron concentrations. This may suggest that in individuals with a BMI of 25–30 kg/m^2^, moderate iron levels provide protection, whereas higher iron levels may increase the risk of ASCVD. On the other hand, for individuals with a BMI over 30 kg/m^2^, their bodies may have adapted to higher oxidative stress, leading to a consistent negative association between serum iron levels and ASCVD across a broader range of iron levels. Consistent with our findings, a study conducted at a Chinese hospital with 221 patients, found that both low and high serum iron levels were linked to increased odds of Major Adverse Cardiovascular and Cerebrovascular Events (MACCE) (24). Additionally, there is also a certain correlation between BMI and serum iron levels. Research indicates that a higher BMI is typically associated with lower serum iron levels (25), which aligns with the results of our current study.

In our research, we focused on serum iron levels instead of ferritin to assess the relationship with ASCVD. Ferritin is known to be an indicator of inflammation (26), which can make it an unreliable measure of iron status in chronic inflammatory conditions like ASCVD. In contrast, while serum iron levels are also influenced by inflammation, they are less affected compared to ferritin and more accurately reflect the available iron in the blood. Therefore, serum iron serves as a more precise marker for understanding iron’s role in the development of ASCVD. Among the biomarkers of iron metabolism, serum iron has been extensively studied in relation to predicting adverse events (24). Notably, low serum iron levels, irrespective of ferritin or hemoglobin levels, have been associated with increased all-cause mortality in heart failure patients (27).

Iron is an essential micronutrient crucial for various biological processes, including enzymatic activities, mitochondrial function, and energy metabolism (28, 29). During exercise or periods of increased heart rate, iron deficiency can negatively impact cardiac performance (30, 31). As a vital cofactor for antioxidant enzymes such as catalase and cytochrome oxidase (32), iron deficiency hampers the production of these enzymes. This deficiency can lead to oxidative damage and endothelial dysfunction (22), and it can also inactivate antithrombin via the thrombin/Factor XII complex, promoting a hypercoagulable state (33). Consequently, maintaining adequate iron levels may reduce the risk of ASCVD.

Iron’s ability to change valence states enables it to efficiently transport O_2_ and facilitate electron transfer, which can lead to the generation of ROS (34). Iron overload increases oxidative stress through Fenton reactions and the upregulation of oxidases (23), ultimately promoting the progression of AS (35). In 1981, Jerome Sullivan introduced the “iron hypothesis,” suggesting that excess iron might contribute to cardiovascular damage and that reducing iron levels could potentially protect against CVDs (36). The concept of ferroptosis, introduced in 2012 (37), has gained increasing recognition, with research confirming its role in ASCVD (38). For individuals at high risk for ASCVD, iron depletion to near-deficiency (NID) appears to be a more favorable metabolic state than iron sufficiency (39).

Gender significantly influences the risk of ASCVD. Since 1984, women have consistently exhibited higher annual mortality rates from CVD compared to men (40). In women, high dietary iron intake has been associated with a 65% increase in coronary heart disease (CHD) risk, and this risk remained unaffected by other factors, including additional risk factors, menstruation, or antioxidant intake (41). In a study of 1,992 hypertensive patients, men showed a U-shaped ASCVD risk with serum ferritin levels, whereas women demonstrated a linear increase in ASCVD risk with higher iron levels (42). In our study, gender differences were not pronounced, likely because all participants were over the age of 60. The gender differences observed in other studies might be related to the menstrual cycle, as continuous blood loss during menstruation could make the cardiovascular system more resilient and resistant (43). Additionally, all participants in the referenced study were hypertensive patients, while only 71.32% of our study population had hypertension. The presence of hypertension might influence the association between iron levels and ASCVD risk (42).

Smoking is explicitly identified as a major risk factor for ASCVD (44). It directly damages vascular endothelial function (45), altering iron metabolism and increasing oxidative stress (46), contributing to lipid anomalies, inflammation (45), and thrombosis (47) to exacerbate the risk of ASCVD. ROS and nicotine in cigarette smoke increase oxidative stress, directly impacting iron metabolism and storage (46).

In our analysis, interaction tests revealed no significant factors altering the relationship between serum iron levels and ASCVD, suggesting a consistent association across various subgroups and indicating a level of robustness in this relationship. However, the subgroup analysis identified significant variations across multiple factors, notably including gender, BMI, PIR, and smoking, among others. This suggests that while the overall interaction was not significant, these specific factors may still influence the relationship between serum iron levels and ASCVD risk within certain subgroups. This highlights the need for further investigation into these potential influences. Therefore, these results emphasize the need for individualized treatment strategies, taking into consideration the unique characteristics of each patient, rather than adopting a one-size-fits-all approach to iron management.

Our study has several advantages: (1) Representative Sample: The survey includes a representative sample from diverse racial and ethnic groups, enhancing its generalizability to the US population. The large sample size also provides broader scope and greater statistical power compared to previous studies. (2) Control of Confounding Factors: Acknowledging the potential influence of confounders in observational studies, we implemented rigorous control strategies to minimize residual confounding effects. (3) Stratified and Interaction Analysis: We performed stratified and interaction effect analyses to uncover hidden patterns and relationships within the data. However, it is important to recognize several limitations: (1) Self-Reported Data: The CVD data in the NHANES database were obtained through self-reported questionnaires instead of clinical diagnoses, which could introduce specific biases (48). (2) Cross-Sectional Study Design: The nature of a cross-sectional study limits its ability to establish causality or explore temporal dynamics, focusing instead on identifying correlations (49). (3) Population-Specific Findings: This study was conducted on US individuals, limiting the generalizability of the results to different populations.

## 5 Conclusion

In conclusion, the study suggests that serum iron levels below 131 μg/dL are negatively associated with ASCVD, while the association between higher serum iron levels and ASCVD risk was not statistically significant. This relationship was particularly evident in individuals with a BMI of 25–30 kg/m^2^, where moderate iron levels seemed protective. However, the potential risks associated with higher serum iron levels in this subgroup warrant further investigation. Future longitudinal studies and clinical trials are necessary to clarify the causal relationship and to explore the implications of these findings for clinical practice.

## Data Availability

Publicly available datasets were analyzed in this study. This data can be found here: direct link to the data: https://www.cdc.gov/nchs/nhanes/index.htm.

## Footnotes

1
http://www.R-project.org

2
www.empowerstats.com
