# Supplementary material for: Association between serum iron levels and atherosclerotic cardiovascular diseases among American older adults: a cross-sectional study based on the National Health and Nutrition Examination Survey, 2009–2018
Source: Front Nutr. 2024 Sep 25;11:1457236. doi: 10.3389/fnut.2024.1457236 (PMC11463155; doi:10.3389/fnut.2024.1457236)
Supplement: Supplementary file 1 [file Table_1.DOCX]

Supplementary Table 1. Disease diagnosis from NHANES database.

| **Disease** | **Section** | **English Text** | **Value Description for Diagnosis Yes** |
| --- | --- | --- | --- |
| coronary heart disease | Questionnaire Data | Has a doctor or other health professional ever told {you/SP} that {you/s/he} . . .had coronary (kor-o-nare-ee) heart disease? | Yes |
| angina | Questionnaire Data | Has a doctor or other health professional ever told {you/SP} that {you/s/he} . . .had angina (an-gi-na), also called angina pectoris? | Yes |
| heart attack | Questionnaire Data | Has a doctor or other health professional ever told {you/SP} that {you/s/he} . . .had a heart attack (also called myocardial infarction (my-o-car-dee-al in-fark-shun))? | Yes |
| stroke | Questionnaire Data | Has a doctor or other health professional ever told {you/SP} that {you/s/he} . . .had a stroke? | Yes |
| diabetes | Questionnaire Data | The next questions are about specific medical conditions. {Other than during pregnancy, {have you/has SP}/ {Have you/Has SP}} ever been told by a doctor or health professional that {you have/{he/she/SP} has} diabetes or sugar diabetes? | Yes |
|  | Laboratory Data | Glycohemoglobin | ≥6.5% |
|  | Laboratory Data | Fasting glucose | ≥126mg/dl |
|  | Laboratory Data | random blood glucose | ≥200mg/dl |
|  | Laboratory Data | Two Hour Glucose (OGTT) | ≥200mg/dl |
| hyperlipidemia | Questionnaire Data | To lower (your/his/her) blood cholesterol, (have/has) (you/SP) ever been told by a doctor or other health professional ... to take prescribed medicine? | Yes |
|  | Laboratory Data | TG | ≥150 mg/dL |
|  | Laboratory Data | TC | ≥200 mg/dL |
|  | Laboratory Data | LDL-C | ≥130 mg/dL |
|  | Laboratory Data | HDL-C | ≤40mg/dL(male), ≤50mg/dL (female) |
| hypertension | Questionnaire Data | {Have you/Has SP} ever been told by a doctor or other health professional that {you/s/he} had hypertension, also called high blood pressure? | Yes |
|  | Questionnaire Data | {Were you/Was SP} told on 2 or more different visits that {you/s/he} had hypertension, also called high blood pressure? | Yes |
|  | Questionnaire Data | Because of {your/SP's} (high blood pressure/hypertension), {have you/has s/he} ever been told to . . . take prescribed medicine? | Yes |
|  | Questionnaire Data | HELP AVAILABLE (Are you/Is SP) now taking prescribed medicine | Yes |
|  | Examination Data | Average SBP, average DBP (at least 3 times) | Average SBP≥140 mmHg,  average DBP≥90 mmHg |
